# Amblyopia screening: a new screening protocol implemented in Croatia

**DOI:** 10.3325/cmj.2016.57.4

**Published:** 2016-02

**Authors:** Mladen Bušić, Mirjana Bjeloš, Biljana Kuzmanović Elabjer

**Affiliations:** University Eye Clinic, University Hospital “Sveti Duh,” Zagreb, Croatia; Faculty of Medicine, Josip Juraj Strossmayer University of Osijek, Osijek, Croatia *mbjelos@mef.hr*

In 1968 Wilson and Jungner defined 10 criteria for a screening program, which have been only slightly changed in the past forty years (1). These criteria were confirmed and expanded by a bulletin of World Health Organization (WHO) published in April 2008 (2). Every screening protocol proposed to be introduced as a policy is not just a matter of medicine and science, but there are political, economic, and ethical issues that need to be addressed before adopting these methods. Human genome sequencing has raised many concerns – in medical and scientific aspect it has enabled screening for various diseases and syndromes, but ethically and economically these methods may prove to be questionable or unfeasible (2). Wilson and Jungner defined screening as “a cross-sectional, short-term operation on a population at risk” (1). Hence, it is necessary to differentiate between examining and treating a single patient from trying to “examine and treat” a community (3), the latter being in some way the goal of screening. Sacket et al (3) claim that an introduction of “untested community screening” may cause irreversible damage to the society and cause a permanent loss of “profession’s credibility.” The implementation of any population method must be guided by public needs and values (4), but established on medical and scientific evidence and measures.

In Croatia, three National Preventive Programs were set up – for early detection of breast cancer in 2006, for colorectal cancer in 2007, and for cervical cancer in 2010 (5). However, some problems in conducting and implementing these screening strategies have been reported (5-7), mainly related to compliance, lack of population education, and organizational issues. For example, in colorectal cancer program, 84% of population at risk received the test package to their home address and only 19% returned the test sample, while the population from islands had difficulties in reaching a center were colonoscopy was performed (6). Another important question was raised concerning the organization and quality of the entire health care system, disclosing some of its weak points, like unequal quality of colonoscopy performance and unwillingness of some physicians to participate in such programs (6). The breast cancer screening program had a response rate of 49%. It proved its major benefit in diagnosing breast cancer of lower stages compared to unscreened population (7). Expectations from cervical cancer screening program are high as Croatia has well-organized infrastructure for this kind of screening and a long tradition of education of cytologists and primary gynecologists (8).

For pediatric population, three screening programs were approved as national health policy in Croatia by June 1, 2015. These included screening for phenylketonuria, conducted in 1978, for congenital hypothyroidism in 1985, and for hearing disorders in 2002. Phenylketonuria screening program in Croatia discovers 5-6 patients per year (9). Although this number does not seem high, early detection of phenylketonuria has a substantial impact not only on affected individuals` lives, but also on society, economy, and labor productivity (10). The neighboring countries have similar national preventive policies for newborns (11).

Children’s screening programs have a major sociological significance because they may bring about a lifetime benefit. Community is very sensitive to the child population, but it has little or no awareness of the disorders. In conclusion, screening programs in childhood that are to be introduced need a thorough medical, scientific, ethic, economic, and sociologic evaluation.

Amblyopia, subnormal visual acuity, is a disorder that meets all the WHO criteria for a screening program (12,13). The gold standard to diagnose amblyopia is a complete ophthalmological examination, however, being an elaborate procedure it cannot be used as a screening method. Several simple and effective screening programs have been introduced since the Wilson and Jungner’s work. However, there is no consensus on the preferred, validated, and effective amblyopia screening protocol. Zagreb Amblyopia Preschool Screening (ZAPS) study aimed at validation and testing of a highly structured protocol, designed to be both highly sensitive and specific, for measuring near and distance visual acuity and raising a threshold value of visual acuity for referral (14). The protocol was conducted and tested in Zagreb, Croatia, on 15 648 children aged 48-54 months attending Zagreb kindergartens. ZAPS study protocol proved to have a high testability rate, sensitivity of 100%, and specificity of 96.68%. By testing visual acuity using optotypes in lines, the prevalence of amblyopia was found to be 8.08%, substantially higher than documented in the current literature. Founded on the evidence that amblyopia screening meets all the WHO criteria for screening program and having ZAPS study protocol as a valid screening tool, the Croatian Ministry of Health in June 2015 adopted a new screening program as an obligatory national health policy addressing all ethical, economic, and political issues, along with social community priorities (15). Pediatricians now refer all four-year-old children to the ophthalmologist, who performs screening examination in accordance with ZAPS study protocol. As stated by Wilson and Jungner, vision screening programs are the hallmark of a country’s development, which places Croatia among the most responsible societies in terms of promotion and enhancement of the quality of children’s health care (1).
